# Heated tobacco products and circulating high-density lipoprotein cholesterol concentrations

**DOI:** 10.1038/s41598-022-22337-3

**Published:** 2022-10-17

**Authors:** Huan Hu, Tohru Nakagawa, Toru Honda, Shuichiro Yamamoto, Toshiaki Miyamoto, Hiroko Okazaki, Masafumi Eguchi, Taiki Shirasaka, Takeshi Kochi, Isamu Kabe, Aki Tomizawa, Takako Miki, Ami Fukunaga, Shohei Yamamoto, Yosuke Inoue, Maki Konishi, Haruka Miyake, Seitaro Dohi, Tetsuya Mizoue

**Affiliations:** 1 Research Center for Prevention From Radiation Hazards of Workers, National Institute of Occupational Safety and Health, Kanagawa, Japan; 2 Department of Epidemiology and Prevention, National Center for Global Health and Medicine, Tokyo, Japan; 3 Hitachi Health Care Center, Hitachi, Ltd., Ibaraki, Japan; 4 Nippon Steel Corporation, East Nippon Works Kimitsu Area, Chiba, Japan; 5 Mitsui Chemicals, Inc., Tokyo, Japan; 6 Furukawa Electric Co., Ltd., Tokyo, Japan; 7 KUBOTA Corporation Co., Ltd., Ibaraki, Japan; 8 Health Design Inc., Tokyo, Japan

**Keywords:** Risk factors, Disease prevention, Occupational health, Public health

## Abstract

We aimed to assess the association between heated tobacco product (HTP) use and high-density lipoprotein cholesterol (HDL-C) concentration. Our study included 12,268 workers from five companies (Study I) and 36,503 workers from another large company (Study II). Participants were categorized into five groups: never smokers, past smokers, exclusive HTP users, dual users of cigarettes and HTPs, and exclusive cigarette smokers. We analyzed the data of Studies I and II separately and then pooled these estimates using a fixed-effect model. Of the 48,771 participants, 9.3% were exclusive HTP users, and 6.0% were dual users. Exclusive HTP users had modestly but significantly lower concentrations of HDL-C than never smokers, with the pooled mean difference being − 1.1 (95% CI − 1.5 to − 0.6) mg/dL. Dual users showed a further reduction (mean difference − 3.7 (− 4.2 to − 3.2) mg/dL), which was comparable to that of exclusive cigarette smokers versus never smokers (mean difference − 4.3 (− 4.7 to − 3.9) mg/dL). The pooled odds ratios (95% CIs) of having low HDL-C (< 40 mg/dL for men and 50 mg/dL for women) were 1, 0.99 (0.90–1.11), 1.25 (1.09–1.43), 2.02 (1.76–2.32), and 2.09 (1.88–2.32) for never smokers, past smokers, exclusive HTP users, dual users, and exclusive cigarette smokers, respectively. In conclusion, exclusive HTP users had lower HDL-C concentrations than never smokers, although higher than exclusive cigarette smokers. Moreover, dual users had HDL-C concentrations similar to those in exclusive cigarette smokers.

## Introduction

There is a growing popularity for the use of heated tobacco products (HTPs), which were originally proposed to be less harmful alternatives to conventional cigarettes^1^. Currently, these products are available in more than 40 countries^2^. Japan has been the testbed for HTPs since they were marketed in 2014^3^, with 11% of people aged 15–74 years being users of these products in 2020^4^. A review of chemical evidence showed that the harmful and potentially harmful constituents in HTPs are the same as in conventional cigarette smoke, albeit in lower concentrations^5^. However, the studies assessing the health impact of HTP use are rare, and most of them were funded by the tobacco industry^6^.

In terms of cardiovascular health, the link between HTPs and circulating high-density lipoprotein cholesterol (HDL-C), a sensitive marker of cigarette smoking^7^ and predictor of cardiovascular disease (CVD)^8^, is of interest. An industry-sponsored trial showed that cigarette smokers who switched to HTPs had higher HDL-C concentrations than those who continued cigarette smoking at month 6^9^. Likewise, an industry-sponsored cross-sectional study showed that exclusive HTP users had higher HDL-C concentrations than cigarette smokers^10^. To our knowledge, however, there have been no published investigator-initiated population-based studies on this issue. Additionally, evidence on the effect of the dual use of cigarettes and HTPs is lacking. In this study, we examined the association between HTP use and HDL-C concentration using data from a large working population in Japan.

## Methods

### Setting

We used data from the Japan Epidemiology Collaboration on Occupational Health (J-ECOH) study, an ongoing multi-company study of workers. In Japan, workers are obliged to undergo health check-ups at least once a year under the Industrial Safety and Health Act. The participants in the J-ECOH study underwent annual health check-ups, including a self-administered questionnaire, physical examinations, and laboratory examinations, as described elsewhere^11^. In Phase 3, between April 2018 and March 2021, five companies participated in a questionnaire survey on the use of new tobacco products (Study I). In another large company, information on the use of new tobacco products was available from 2019 health check-ups (study II). This study was performed in accordance with the ethical standards of the 1975 Declaration of Helsinki, as revised in 2013. The study protocol was approved by the ethics committee of the National Center for Global Health and Medicine, Japan (NCGM-G-001140). Informed consent was obtained from all participants.

### Participants

#### Study I

A total of 12,846 workers completed the questionnaires. Of these, 12,672 had health check-up data for the same fiscal year as the questionnaire survey. We excluded individuals who had missing data on smoking (n = 17), reported smoking e-cigarettes (n = 116), or lacked data on HDL-C or covariates (n = 117). In one company, where the questionnaire survey was conducted more than six months after the health check-up of that fiscal year, we excluded 154 people who reported having quit cigarette smoking in the past year to reduce the potential carry-over effects of past smoking and misclassification. This left 12,268 participants for the analysis.

#### Study II

Of the 49,333 workers who attended the 2019 health check-up, we excluded those who had missing data on smoking (n = 8090), HDL-C (n = 393), or covariates (n = 4347), leaving 36,503 participants for the analysis.

### Exposure

In Study I, cigarette smoking was identified by the question “Do you smoke conventional cigarettes?” Participants were asked to select one of the following options: never, quit ≥ 5 years ago, quit < 5 years ago, smoke 1–5, 6–10, 11–20, and > 20 cigarettes per day. New tobacco product use was identified by the question, “Did you use new tobacco products in the last month?” The participants were asked to select one or more of the following answers: no, used HTPs (e.g., IQOS, Ploom Tech, glo), and used e-cigarettes. We excluded e-cigarette dual users (n = 46) and exclusive users (n = 70) because of their small sample size. The remaining participants were divided into five groups: never smokers, past smokers, exclusive HTP users, dual users of conventional cigarettes and HTPs, and exclusive conventional cigarette smokers.

Study II used a slightly different questionnaire. The above five groups were identified based on their responses to the following questions: (1) Are you a current smoker? (never smoker, past smoker, and current smoker); for current smokers, (2) Which tobacco products do you use? (conventional cigarettes only, new tobacco products only (e-cigarettes and/or HTPs), and both conventional and new tobacco products). In addition, participants were also asked how many cigarettes/HTPs they used per day. The use of e-cigarettes is low in Japan^12^, and only 5% of the new tobacco product users reported using e-cigarettes in Study I. Thus, ‘new tobacco products’ in Study II were treated as HTPs in the analysis.

### Outcome

The HDL-C concentration was measured using an enzymatic method. Low HDL-C was defined as HDL-C concentration < 40 mg/dL for men and 50 mg/dL for women^13^.

### Covariates

The covariates included age, sex, body mass index (BMI), alcohol consumption, and leisure-time physical activity. Body height and weight were measured using a scale while participants wore light clothes and no shoes. BMI was calculated as weight in kilograms divided by the square of height in meters. Average daily alcohol consumption was calculated as the drinking frequency multiplied by ethanol consumption per drinking day. In Study I, leisure-time physical activity was expressed as the sum of the metabolic equivalent (MET) multiplied by the duration of time (in h) across physical activities with three different intensity levels. In Study II, the weekly minutes of leisure-time physical activity were calculated as the frequency of physical activity multiplied by the duration of the activity engaged per day and summed up to 20 activities, as described elsewhere^14^.

### Statistical analysis

The characteristics of the study participants are described as means and standard deviations for continuous variables and percentages for categorical variables. We performed multiple linear regression to estimate the means and 95% confidence intervals (CIs) of HDL-C concentrations according to smoking groups, with adjustment for age, sex, BMI (kg/m^2^), alcohol consumption (non-drinker, drinkers consuming < 1, 1 to < 2, or ≥ 2 *go*/day; one *go* contains approximately 23 g of ethanol), and leisure-time physical activity (Study I: 0, > 0 to < 3, 3 to < 10, or ≥ 10 MET-h/week; Study II: < 150 min/week or ≥ 150 min/week). The worksite was treated as a cluster factor. Additionally, we performed logistic regression analysis to estimate the odds ratios (ORs) and 95% CIs for low HDL-C levels associated with tobacco product use, using never smokers as the reference group. We analyzed the data of Studies I and II separately and then pooled the estimates using a fixed-effect model, considering the similarity of the two studies in terms of study design and population. We confirmed no material differences in the results using the random-effect model. Further, we conducted a dose–response analysis using data from Study II. Exclusive HTP users, dual users, and exclusive cigarette smokers were divided into 3 groups based on the number (1–9, 10–19, ≥ 20) of cigarettes/HTPs they used per day. We analyzed individual-level data using SAS version 9.4 (SAS Institute, Cary, NC, USA) and performed a meta-analysis using STATA version 14 (StataCorp, College Station, TX, USA). A two-sided P value of < 0.05 was considered statistically significant.

## Results

Among the 48,771 participants included in the analysis, 82.5% were men, and the mean age was 45.5 ± 11.4 years. Of the total participants, 9.3% exclusively used HTPs, 6.0% used HTPs, and concurrently smoked cigarettes. As shown in Table 1, exclusive HTP users and cigarette smokers had similar characteristics, except that exclusive HTP users were younger. When compared with never smokers, exclusive HTP users were more likely to be male, consume alcoholic drinks, and have higher BMI values.

**Table 1:** Characteristics of study participants.

	Never smoker	Past smoker	Current use of tobacco products		
			Exclusive HTP user	Dual user	Exclusive cigarette smoker
**Study I**					
N	6070	2944	675	1047	1532
Age (years)	40.0 ± 12.5	49.3 ± 9.9	41.8 ± 10.7	39.9 ± 11.4	44.6 ± 11.7
Male (%)	78.6	96.5	97.5	97.2	97.0
Body mass index (kg/m^2^)	23.1 ± 3.7	24.1 ± 3.3	24.1 ± 3.7	24.2 ± 3.9	23.9 ± 3.7
Current alcohol consumption (%)	68.3	82.7	78.1	79.7	78.9
Leisure time physical activity (≥ 10 METs-h/week) (%)	27.3	31.6	24.2	24.6	22.3
**Study II**					
N	17,353	8724	3832	1859	4735
Age (years)	44.2 ± 11.9	50.3 ± 9.3	45.8 ± 8.8	45.3 ± 10.0	48.2 ± 9.6
Male (%)	70.2	92.9	94.5	94.6	93.6
Body mass index (kg/m^2^)	23.5 ± 3.9	24.2 ± 3.5	24.4 ± 3.9	24.6 ± 4.1	24.0 ± 3.8
Current alcohol consumption (%)	51.3	64.2	68.7	68.4	66.8
Leisure time physical activity (≥ 150 min/week) (%)	14.2	19.6	15.9	13.0	12.7

**Figure 1:**
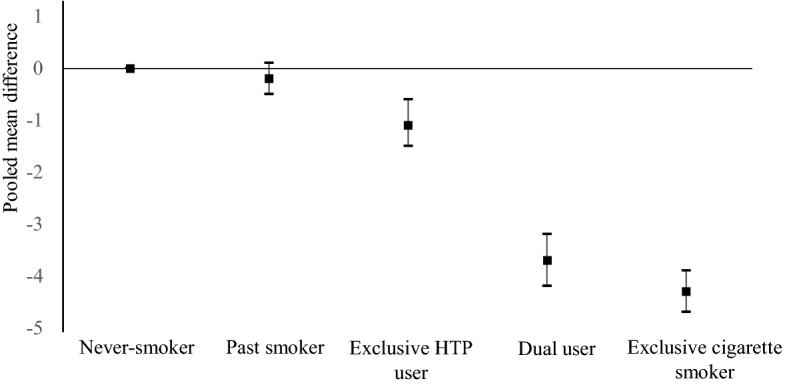
Pooled mean differences (95% confidence intervals) in HDL-C (mg/dL).

Figure 1 and Supplementary Table S1 show that exclusive HTP users had modestly but significantly lower HDL-C concentrations than never smokers, with a pooled mean difference of − 1.1 (95% CI − 1.5 to − 0.6) mg/dL. Dual users showed a further decrease in HDL-C concentrations (pooled mean difference versus never smokers − 3.7 [− 4.2 to − 3.2] mg/dL), which was comparable to that of exclusive cigarette smokers (pooled mean difference vs. never smokers − 4.3 [− 4.7 to − 3.9] mg/dL). We observed no obvious sex difference in the association between tobacco use and HDL-C concentrations (Table S1). As shown in the Supplementary Table S2, for every 10 additional cigarettes/HTPs used per day, there was a decrease of 0.7-mg/dl in HDL-C concentrations among exclusive HTP users, a 2.4-mg/dl decrease for dual users, and a 2.9-mg/dl decrease for exclusive cigarette smokers (all P values for trend < 0.001).

**Figure 2:**
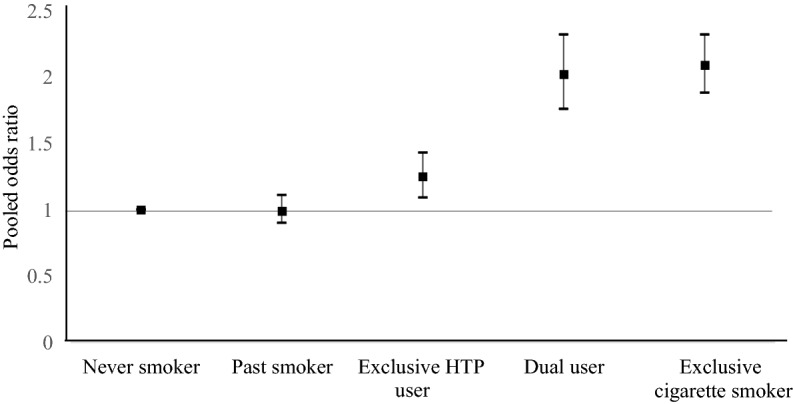
Pooled odds ratios (95% confidence intervals) of low HDL-C associated with the use of tobacco products.

As shown in Fig. 2 and Supplementary Table S3, exclusive HTP users had an increased likelihood of having low HDL-C, compared with never smokers, with a pooled OR of 1.25 (95% CI 1.09–1.43). For dual users, the pooled OR was 2.02 (1.76–2.32), which was similar to that of exclusive cigarette smokers (2.09 [1.88–2.32]).

## Discussion

In this large-scale study among workers, we compared the harmful effects of HTPs and cigarettes on HDL-C and found that the HDL-C concentration among exclusive HTP users fell between never smokers and exclusive cigarette smokers. Moreover, HDL-C concentrations among dual users were comparable to those of cigarette smokers. To our knowledge, this is one of the few studies on the association between HTP use and HDL-C concentrations.

Our finding of higher HDL-C concentrations among exclusive HTP users than among exclusive cigarette smokers is consistent with previous studies^9,10^. An industry-sponsored trial among 984 American smokers reported that the HDL-C level was 3 mg/dL higher in the HTP use group than in the continued cigarette smoking group^9^. In addition, an industry-sponsored cross-sectional study of 459 Japanese individuals reported that exclusive HTP users had about 7 mg/dL higher HDL-C than cigarette smokers, with adjustment for age, sex, and BMI^10^. Our study showed a difference of 3 mg/dL in HDL-C between exclusive HTP users and cigarette smokers after adjusting for a wide range of covariates, including age, sex, BMI, alcohol consumption, and leisure-time physical activity. Moreover, we found a slight but statistically significant difference in HDL-C levels between HTP users and never smokers (− 1.1 mg/dL). These findings suggest that HTP use is associated with lower HDL-C levels, although the reduction was smaller than that of conventional cigarette use. Compared with cigarettes, HTP aerosols contain lower levels of harmful toxicants, such as aldehydes and nicotine^15^, which can affect HDL-C metabolism and its subfraction distribution by reducing lecithin: cholesterol acyltransferase activity, altering cholesteryl ester transfer protein, and hepatic lipase activity^16,17^.

In our study, 40% of the HTP users smoked cigarettes concurrently. In the Japan “Society and New Tobacco” Internet Survey, over 60% of HTP users smoked cigarettes in 2019^18^. We found that dual users had a 3.7 mg/dL lower mean HDL-C concentration than never smokers, and the reduction was comparable to that of exclusive cigarette smokers (4.3 mg/dL lower than never smokers). In Study II, in which detailed data on cigarette consumption were available, we observed no obvious difference in the mean number of cigarettes smoked per day between dual users (16 cigarettes per day) and exclusive cigarette smokers (15 cigarettes per day). This may partly explain the small difference in HDL-C concentrations between dual and exclusive cigarette smokers. Our data suggest that the concurrent use of cigarettes and HTPs may not help continued smokers decrease cigarette consumption, thus not contributing to the improvement in HDL-C concentrations.

Our results may be of clinical importance. For example, an increase of 1 mg/dL in HDL-C has been linked to a 2–3% decrease in the risk of coronary heart disease^19^. In the present study, we found that the HDL-C level was 3 mg/dL higher in exclusive HTP users than in exclusive cigarette smokers. If this is the case, for people who have difficulty quitting smoking, switching completely from cigarettes to HTPs could lead to a 6–9% decrease in the risk of developing coronary heart disease through an increase in HDL-C. It should be stressed, however, that HTPs are not safe or risk free in light of accumulating evidence that points to a wide range of harmful substances being emitted from HTPs similar to cigarette smoke, albeit at lower levels^5^. Non-cigarette smokers who use HTPs may also have increased risk of developing tobacco-related disorders, given that aerosols from HTPs contain nicotine and other substances that are addictive and can cause harmful effects on health.

The strengths of our study include the well-powered comparison groups of never, past, and current smokers and comprehensive covariate adjustment (e.g., exercise and alcohol consumption). However, our study has some limitations. First, the cross-sectional design of this study did not permit us to establish causality between HTP use and HDL-C concentrations. Second, smoking habits were ascertained via a questionnaire but not biologically verified (e.g., exhaled carbon monoxide or salivary cotinine). Finally, our study is conducted in real-world settings and participants were employees from several large companies in Japan. Thus, our data should be a reflection of the actual use of these products in the Japanese working population, but may not accurately represent other populations, especially in other countries.

## Conclusions

In this large population-based study, users of only HTPs had lower HDL-C concentrations than never smokers, although higher than exclusive cigarette smokers. Individuals who used both HTPs and cigarettes had HDL-C concentrations similar to those of exclusive cigarette smokers. Further studies should investigate whether switching from conventional cigarettes to HTPs decreases CVD risk.

## Supplementary Information

Supplementary Tables.

## Data Availability

The datasets are not publicly available due to privacy/ethical reasons but are available from Dr Tetsuya Mizoue on reasonable request.
