# Supplementary material for: Heated tobacco products and circulating high-density lipoprotein cholesterol concentrations
Source: Sci Rep. 2022 Oct 17;12:17385. doi: 10.1038/s41598-022-22337-3 (PMC9576675; doi:10.1038/s41598-022-22337-3)
Supplement: Supplementary file 1 — Supplementary Tables. [file 41598_2022_22337_MOESM1_ESM.docx]

**Supplementary Appendix**

**Heated tobacco products and circulating high-density lipoprotein cholesterol concentrations**

Huan Hu, Tohru Nakagawa, Toru Honda, Shuichiro Yamamoto, Toshiaki Miyamoto, Hiroko Okazaki, Masafumi Eguchi, Taiki Shirasaka, Takeshi Kochi, Isamu Kabe, Aki Tomizawa, Takako Miki, Ami Fukunaga, Shohei Yamamoto, Yosuke Inoue, Maki Konishi, Haruka Miyake, Seitaro Dohi, Tetsuya Mizoue

Table S1 Mean difference (95% confidence interval) in HDL-C (md/dL) compared to never smokers^a^

|  |  |  | Current use of tobacco products | | | P for interaction between sex and tobacco use | Heterogeneity^b^ P, I^2^ (%) |
| --- | --- | --- | --- | --- | --- | --- | --- |
|  | Never smoker | Past smoker | Exclusive HTP user | Dual user | Exclusive cigarette smoker |  |  |
| **Study Ⅰ** |  |  |  |  |  |  |  |
| Men | 4,770 | 2,841 | 658 | 1,018 | 1,486 | 0.8 |  |
|  | Reference | 0.4 (-0.2 to 1.1) | -1.0 (-2.1 to 0.0) | -2.9 (-3.8 to -2.0) | -3.8 (-4.5 to -3.0) |  |  |
| Women | 1,300 | 103 | 17 | 29 | 46 |  |  |
|  | Reference | -0.9 (-3.9 to 2.1) | -2.4 (-9.5 to 4.7) | -3.7 (-9.3 to 1.9) | -3.1 (-7.5 to 1.2) |  |  |
| Overall | Reference | 0.3 (-0.4 to 1.0) | -1.1 (-2.2 to 0.1) | -2.9 (-3.9 to -2.0) | -3.8 (-4.6 to -2.9) |  |  |
| **Study Ⅱ** |  |  |  |  |  |  |  |
| Men | 1,2189 | 8,112 | 3,621 | 1,758 | 4,434 | 0.7 |  |
|  | Reference | -0.3 (-0.7 to 0.1) | -1.1 (-1.6 to -0.6) | -4.2 (-4.9 to -3.5) | -4.4 (-4.9 to -3.9) |  |  |
| Women | 5,164 | 612 | 211 | 101 | 301 |  |  |
|  | Reference | -0.1 (-1.4 to 1.2) | -0.6 (-2.7 to 1.5) | -3.3 (-6.3 to -0.3) | -4.4 (-6.3 to -2.7) |  |  |
| Overall | Reference | -0.4 (-0.8 to -0.1) | -1.0 (-1.5 to -0.6) | -4.1 (-4.8 to -3.5) | -4.5 (-4.9 to -4.0) |  |  |
| **Pooled results** | | | | | | | |
| Men | Reference | -0.1 (-0.4 to 0.3) | -1.1 (-1.5 to -0.6) | -3.7 (-4.3 to -3.2) | -4.2 (-4.6 to -3.8) |  | 0.9, 0 |
| Women | Reference | -0.2 (-1.4 to 1.0) | -0.8 (-2.8 to 1.3) | -3.4 (-6.1 to -0.8) | -4.3 (-5.9 to -2.6) |  | 0.6, 0 |
| Overall | Reference | -0.2 (-0.5 to 0.1) | -1.1 (-1.5 to -0.6) | -3.7 (-4.2 to -3.2) | -4.3 (-4.7 to -3.9) |  | 0.9, 0 |

^a^ Adjusted for age, sex, body mass index, alcohol consumption, and leisure time physical activity.

^b^ For the Exclusive HTP user group

Table S2 Dose-response relationship between tobacco use and HDL-C (md/dL)^a^

|  |  | Number of cigarettes / HTPs used per day | | | *P* for trend^b^ | Linear trend^c^ |
| --- | --- | --- | --- | --- | --- | --- |
|  | Never smoker | 1-9 | 10-19 | ≥20 |  |  |
| Exclusive HTP user | 17,353 | 294 | 2,053 | 1,467 |  |  |
|  | Reference | -0.4 (-2.0 to 1.2) | -1.3 (-1.9 to -0.7) | -0.9 (-1.7 to -0.2) | P<0.001 | -0.7 (-1.0 to -0.4) |
| Dual user | 17,353 | 91 | 903 | 857 |  |  |
|  | Reference | -2.7 (-5.5 to 0.1) | -4.0 (-4.9 to -3.1) | -4.4 (-5.4 to -3.5) | P<0.001 | -2.4 (-2.8 to -2.0) |
| Exclusive cigarette smoker | 17,353 | 438 | 2,319 | 1,952 |  |  |
|  | Reference | -1.3 (-2.6 to 0.0) | -3.9 (-4.5 to -3.3) | -6.3 (-6.9 to -5.6) | P<0.001 | -2.9 (-3.2 to -2.7) |

^a^ Adjusted for age, sex, body mass index, alcohol consumption, and leisure time physical activity.

^b^ *P* for trend calculated using number of cigarettes / HTPs used per day as a continuous variable.

^c^ Estimated based on every 10 cigarettes/HTPs per day increment.

Table S3 Odds ratio (95% confidence interval) of low HDL-C associated with the use of tobacco products^a^

|  |  |  | Current use of tobacco products | | | Heterogeneity^b^ P, I^2^ (%) |
| --- | --- | --- | --- | --- | --- | --- |
|  | Never smoker | Past smoker | Exclusive HTP user | Dual user | Exclusive cigarette smoker |  |
| **Study Ⅰ** |  |  |  |  |  |  |
| Cases/people | 355/6,070 | 149/2,944 | 54/675 | 109/1,047 | 150/1,532 |  |
| Multivariate-adjusted model | Reference | 1.00 (0.81 to 1.25) | 1.44 (1.05 to 1.98) | 1.99 (1.56 to 2.55) | 2.05 (1.64 to 2.55) |  |
| **Study Ⅱ** |  |  |  |  |  |  |
| Cases/people | 1,120/17,353 | 447/8,724 | 257/3,832 | 204/1,859 | 490/4,735 |  |
| Multivariate-adjusted model | Reference | 0.99 (0.89 to 1.10) | 1.21 (1.04 to 1.40) | 2.04 (1.72 to 2.41) | 2.10 (1.86 to 2.37) |  |
| **Pooled results** | Reference | 0.99 (0.90 to 1.11) | 1.25 (1.09 to 1.43) | 2.02 (1.76 to 2.32) | 2.09 (1.88 to 2.32) | 0.3, 0 |

^a^ Multivariate-adjusted model, adjusted for age, sex, body mass index, alcohol consumption, and leisure time physical activity.

^b^ For the Exclusive HTP user group
